# Systematic analysis of video data from different human–robot interaction studies: a categorization of social signals during error situations

**DOI:** 10.3389/fpsyg.2015.00931

**Published:** 2015-07-08

**Authors:** Manuel Giuliani, Nicole Mirnig, Gerald Stollnberger, Susanne Stadler, Roland Buchner, Manfred Tscheligi

**Affiliations:** Department of Computer Sciences, Center for Human-Computer Interaction, University of SalzburgSalzburg, Austria

**Keywords:** social signals, error situation, social norm violation, technical failure, human–robot interaction, video analysis

## Abstract

Human–robot interactions are often affected by error situations that are caused by either the robot or the human. Therefore, robots would profit from the ability to recognize when error situations occur. We investigated the verbal and non-verbal social signals that humans show when error situations occur in human–robot interaction experiments. For that, we analyzed 201 videos of five human–robot interaction user studies with varying tasks from four independent projects. The analysis shows that there are two types of error situations: social norm violations and technical failures. Social norm violations are situations in which the robot does not adhere to the underlying social script of the interaction. Technical failures are caused by technical shortcomings of the robot. The results of the video analysis show that the study participants use many head movements and very few gestures, but they often smile, when in an error situation with the robot. Another result is that the participants sometimes stop moving at the beginning of error situations. We also found that the participants talked more in the case of social norm violations and less during technical failures. Finally, the participants use fewer non-verbal social signals (for example smiling, nodding, and head shaking), when they are interacting with the robot alone and no experimenter or other human is present. The results suggest that participants do not see the robot as a social interaction partner with comparable communication skills. Our findings have implications for builders and evaluators of human–robot interaction systems. The builders need to consider including modules for recognition and classification of head movements to the robot input channels. The evaluators need to make sure that the presence of an experimenter does not skew the results of their user studies.

## 1. Introduction

The interaction between humans and robots is often affected by problems that occur during such interactions. Human users interact with robots based on their mental models, expectations, and previous experiences. When problems occur, users are often confused. Their expectations are violated and they do not know how to react. In the worst case, such problems can even result in a termination of the interaction (Scheutz et al., 2011). The users, most likely, attribute the error to the robot, and these problems are in fact often caused by the robot. Some examples for occurring problems are insufficient or defective sensor data, errors due to misinterpretation of sensor data from the robot's reasoning module, and general implementation errors (Goodrich and Schultz, 2007). In some cases, however, an interruption of the interaction may also be caused by the human, for example, if the human interaction partner wants to perform a task that is not within the capability of the robot. Irrespective of the origin, the human interaction partner gets confused and the continuation of the interaction is at stake.

Ample evidence for the occurrence of the above described problems can be found in the data of human–robot interaction (HRI) user studies, in which humans directly interact with robots. The matter of interest in these studies is often an envisioned flawless interaction. Therefore, data of problematic interactions may get discarded from further analysis or the problem itself is not part of the analysis. We argue that these data potentially bear valuable insights and ideas for improving future HRI. We are interested in the following questions: what are the social signals that humans display in the event of these errors and what kind of error situations do arise in human–robot interactions?

### 1.1. Social signals

The term *social signal* is used to describe verbal and non-verbal signals that humans use in a conversation to communicate their intentions. Vinciarelli et al. (2009) argue that the ability to recognize social signals is crucial to mastering social intelligence. In their view, the recognition of social signals will be the next step toward a more natural human-computer and human–robot interaction. Ekman and Friesen (1969) define five classes of human non-verbal behavior. *Emblems* are gestures that have a meaning for members of a group, class, or culture, e.g., the thumbs up sign that means positive agreement in many western countries. *Illustrators* are gestures or movements that are directly tied to speech and are used to illustrate what has been said verbally, e.g., humans forming a triangle with their fingers while speaking about a triangular-shaped object. *Affect displays* are signals used to convey an emotional state, often by facial expressions or body posture. *Regulators* are signals used to steer the conversation with a conversation partner, e.g., to regulate turn taking. Finally, *adaptors* are actions used on objects in the environment or on oneself, e.g., lip biting or brushing back hair. The social signals that we detected in our video analysis are mostly affect displays, regulators, and adaptors (see Section 3). For annotating social signals, we are not following Ekman's taxonomy. Instead, we separate the signals into the body parts that the participants in the HRI studies used to express the signal, which makes it easier to annotate combinations of social signals (see Section 2.4).

In recent years, more and more researchers worked on the automatic recognition of social signals, an area that is called *social signal processing*. Vinciarelli et al. (2012) give an overview of the field. They classify social signals with a similar taxonomy that we are using in the annotation scheme in Section 2.4. According to Vinciarelli et al. (2009), human social signals come either from physical appearance, gesture and posture, face and eyes behavior, space and environment, or vocal behavior. The authors also review early work from social signal processing. In human–robot interaction, social signal processing also receives more attention by researchers from different areas. Jang et al. (2013) present a video analysis that is similar to our work. In the analysis, they annotated recordings of six one-on-one teacher–student learning sessions, in order to find the social signals with which students signal their engagement in the interaction. The goal of this work is to implement an engagement classifier for a robot teacher. Tseng et al. (2014) present a robot that automatically recognizes the spatial patterns of human groups by analysing their non-verbal social signals in order to appropriately approach the group and offer services.

A second area of interest to HRI, is the generation of social signals by robots. Bohus and Horvitz (2014) presented a direction-giving robot that forecasts when the user wants to conclude the conversation. This robot uses hesitations (e.g., the robot says “so…”) when it is not certain about the user state in order to get more time to compute a correct forecast and also to convey the uncertainty of the robot. Bohus and Horvitz did not report an improvement in disengagement forecasts for their robot which used hesitations. This might have been due to the conservative strategy they were using in their study, which was tuned to avoid false disengagements. Sato and Takeuchi (2014) researched how the eye gaze behavior of a robot can be used to control the turn taking in non-verbal human–robot interactions. In their study, three humans played a game with a robot that was programmed to look at the other players during the game. The study shows that the robot's gaze can influence who will be the next speaker in the conversation. In another eye gaze generation study, Stanton and Stevens (2014) found that robot gaze positively influences the trust of experiment participants who had to give answers to difficult questions in a game, but negatively influences trust when answering easy questions. However, robot gaze positively influences task performance for easy questions, but negatively influences task performance for difficult questions. Stanton and Stevens discuss that robot gaze might put pressure on the experiment participants. Carter et al. (2014) presented a study, in which participants repeatedly threw a ball to a humanoid robot that attempted to catch the ball. In one of the study conditions, when the robot did not catch the ball, it generated social signals, e.g., it shrugged its shoulders. The study results show that participants smile more when the robot displays social signals and rate the robot as more engaging, responsive, and human-like.

### 1.2. Error situations

In the videos of the experiments that we annotated for this work, we found two different kinds of error situations. On one hand, there were situations in which unusual robot behavior led to a violation of a social norm; on the other hand, there were error situations because of technical failures of the robot. In this section, we will review related work on both of these areas to define our notion of the term *error situation*.

We follow the definition of Sunstein (1996) that *social norms* are “social attitudes of approval and disapproval, specifying what ought to be done and what ought not to be done” (Sunstein, 1996, p. 914). Human interaction is defined by social norms. For example, they define how one should ask for directions on the street or how you should behave in a bar. Schank and Abelson (1977) showed that everyday social interactions have an underlying *social script*, a definition of interaction steps to which humans usually obey. The order of these interaction steps is guided by social signals. Loth et al. (2013) found that customers use two combined non-verbal social signals to signal bartenders that they would like to order a drink: they position themselves directly at the bar counter and look at a member of staff. We define a *violation of a social norm* as a deviation from the social script or the usage of the wrong social signals. For example, in our videos there are instances in which the robot executed unexpected actions in the interaction (e.g., asking for directions several times although the human already gave correct instructions and the robot acknowledged the instructions) or showed unusual social signals (e.g., not looking directly at the person it is talking to).

The second class of error situations in our experiment videos arises from *technical failures* of the robot. Interestingly, we can resort to definitions of technical failures of humans interacting with machines, in order to classify these errors, since all robots we observed are autonomous agents. Rasmussen (1982) defines two kinds of human errors: *execution failures* happen when a person carries out an appropriate action, but carries it out incorrectly, and *planning failures* happen when a person correctly carries out an action, but the action is inappropriate. To transfer these definitions to autonomous robots and to make the definitions clearer, consider the following two examples. The robot makes an execution failure, when it picks up an object, but loses it while grasping it; the robot has a planning failure, when the decision mechanism of the robot decides to ask the human for directions, although it already did so and the human correctly gave the information. Execution failures are also called slips or lapses, while planning failures are mistakes^1^.

To summarize these two definitions (further described in Section 3), we found two types of error situations in the videos we annotated. The robots either violated social norms by executing interaction steps at the wrong time or by showing unusual social signals, or they had obvious technical failures. It is interesting to note that social norm violations often arise of planning failures by the robot, while technical failures are usually execution failures.

Social neuroscientists have studied error situations and how they are perceived by humans. Forbes and Grafman (2013) define social neuroscience as “The systematic examination of how social psychological phenomena can be informed by neuroscience methodologies, and how our understanding of neural function can be informed by social psychological research” (Forbes and Grafman, 2013, p. 1). In recent years, several neuroscientists conducted studies to research the neural correlations when humans observe error situations.

Berthoz et al. (2002) conducted a study to find the neural systems that support processing of intentional and unintentional social norm violations. They used event-related functional magnetic resonance imaging (fMRI) to compare the neural responses of humans listening to stories describing either normal behavior, embarrassing anecdotes, or social norm violations. Berthoz et al. found that the neural systems involved in processing social norm violations are the same as systems involved in representing mental states of others and in responding to aversive emotional expressions. The authors conclude that the findings have implications for understanding the pathology of patients who exhibit social behavioral problems associated with the identified neural systems.

de Bruijn et al. (2011) conducted a study to research whether humans represent the task of a co-actor during error monitoring in joint action. The authors showed through measurement of electroencephalogram (EEG) signals and behavioral data that the study participants show increased amplitudes on the response-locked error-related negativity, an event-related brain potential that is generated after an erroneous response (Falkenstein et al., 1990), and longer reaction times following own errors in a social go/no-go task. The findings show that people incorporate the tasks of others into their own error monitoring and adjust their own behavior during joint action.

Radke et al. (2011) investigated brain activities in humans when monitoring errors that only influenced themselves or also had implications for others. They found in an fMRI study that monitoring errors that have implication for others activates the medial prefontal cortex, a part of the mentalizing system. The authors conclude from the results that this for example explains symptoms of patients with obsessive-compulsive disorder, who have fears that their own actions will harm others.

Ridderinkhof et al. (2004) conducted a meta-analysis of primate and human studies as well as of human functional neuroimaging literature. The analysis showed that the detection of unfavorable outcomes, response errors, response conflict, and decision uncertainty enhances brain activity in an extensive part of the posterior medial frontal cortex. This indicates that performance monitoring, including error monitoring, is associated with this brain region. Koban et al. (2013) recently showed in an event-related fMRI study that error monitoring is integrated with the representation of pain of others. The results of their study show that the same brain regions are involved in error monitoring and empathy for pain and that the brain activity in these regions is enhanced when the pain of the other person is caused by oneself.

In this paper, we perform a systematic analysis of video data from different HRI user studies. The first goal for this analysis is to identify those situations in interactions between humans and robots that lead to problems and create error situations. Such problems include long dialogue pauses, repetitions in the dialogue, misunderstandings, and even a complete abruption of the interaction. In the next step, we categorize the detected error situations into problems resulting from social norm violations and problems that occur due to a technical error. Based on this categorization, we analyse the social signals that humans produce during the problematic situation, in order to map situations and social signals. We distinguish verbal and non-verbal social signals, e.g., speech, gaze, head orientation, and body posture.

For the analysis, we use video data from a variety of HRI user studies. The videos were taken from different projects, providing us with a wide spectrum of robots, robot tasks, and experimental settings. The JAMES project (Joint Action for Multimodal Embodied Social Systems^2^) used a stationary bartender robot with social skills, presenting humans with socially appropriate interaction; the JAST project (Joint-Action Science and Technology^3^) used a stationary robot that cooperates with a human in an assembly task; the IURO project (Interactive Urban Robot^4^) used a mobile, wheeled robot that autonomously navigates through densely crowded inner-city environments and actively asks information from pedestrians; and the RPBD project (Robot Programming by Demonstration) used a NAO robot to research kinesthetic robot teaching in an industrial environment.

In our data analysis, we peruse three goals: (1) provide a ranked categorization of social signals including their frequency of occurrence; (2) develop a mapping between error types and social signals in order to understand if there are particular social signals that are typically evoked either by social norm violation or technical failure; and (3) explore the influence of independent variables (e.g., presence of experimenter during the interaction, single vs. group interaction) on the display of social signals.

## 2. Methods and materials

**Figure 1:**
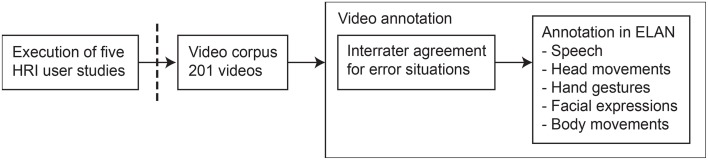
**Chronological steps for the work we carried out in this paper.** First, we executed five human–robot interaction user studies; second, we collected the videos from these studies to form a video corpus; third, we annotated the videos in a two-step process.

Figure 1 shows the work flow of the method that we applied in this paper. First, we executed five HRI user studies^5^, from which we collected a video corpus of 201 interactions. We then annotated the videos in two steps. We introduce the HRI user studies in Section 2.1. Following that, we give an overview of the video corpus in Section 2.2 and information on the study participants in Section 2.3. Finally, we describe the annotation process in more detail in Section 2.4.

### 2.1. Human–robot interaction studies

**Figure 2:**
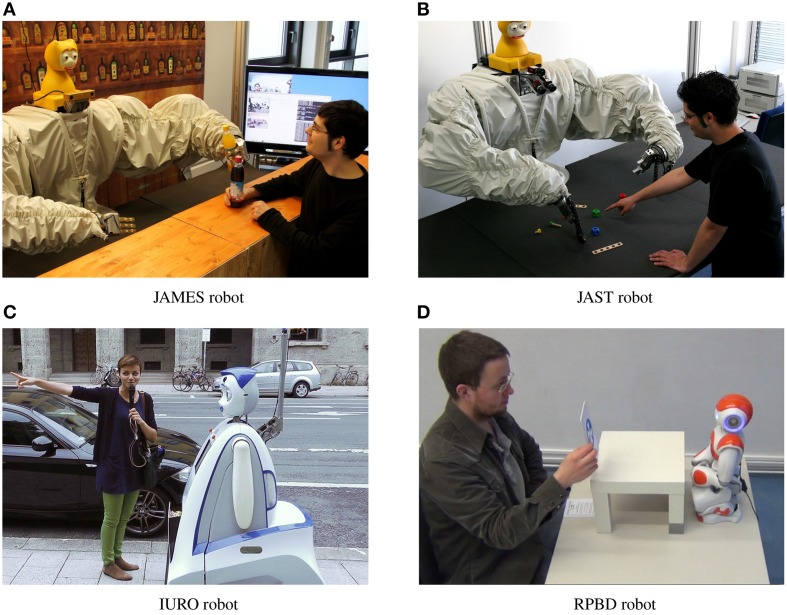
**The four robots used in the human–robot interaction studies.** Pictures show interactions from the studies. **(A)** JAMES robot, **(B)** JAST robot, **(C)** IURO robot, and **(D)** RPBD robot

Our video analysis of social signals in error situations is based on videos from five human–robot interaction studies. These studies were carried out as part of the projects JAMES, JAST, IURO, and RPBD. Each of the studies had a different task for human and robot, except for the two JAMES studies. This enables us to study social signals in the context of a variety of tasks with robots that have different appearances. We have three different humanoid robots (Figure 2), from which one robot is stationary and two robots are mobile. Furthermore, we have four different scenarios, the bartender scenario from JAMES, the joint assembly scenario from JAST, the direction asking scenario from IURO, and the robot teaching scenario from RPBD.

All user studies have in common that the robots were able to understand and produce speech, and that they had visual perception modules for person tracking. The studies were carried out either in Germany or Austria. A majority of the spoken interactions were in German, for the rest human and robot spoke English. We received ethical approval for all of the studies. All study participants signed an informed consent and gave us permission to use the videos taken from the studies for further analysis. The JAMES studies complied to the ethics standards of fortiss (2012, 2013). The JAST study complied to the ethics standards of the Technical University of Munich (2010). The IURO study complied to the Ethics standards of the University of Salzburg (2015). The RPBD study complied to the Ethics standards of the University of Salzburg (2014). For more details on each of the studies, please refer to the publications that we cite for each study in the respective section.

In the following sections we shortly introduce all four projects and describe the user studies from which we used videos. Figure 2 shows images of all four robots.

#### 2.1.1. JAMES, stationary robot bartender

The goal of the JAMES project was to implement successful joint action that is based on social interaction. The task of the JAMES robot was that of a bartender. It had to take drink orders from customers and to hand out the correct drinks to the person who ordered it. Figure 2A shows the robot interacting with a customer. The bartender robot consisted of two industrial robot arms with humanoid hands, mounted in a position to resemble human arms. Furthermore, the robot has an animatronic talking head, the iCat (van Breemen, 2005), which is capable of producing lip-synchronized speech as well as expressing basic facial expressions such as smiling and frowning. The robot was mounted behind a bar, which could be reached by the robot arms. Furthermore, the robot was able to hand over bottles to its customers.

The videos that we are using in this work are from two user studies that were executed with the robot. Foster et al. (2012) researched how the behavior of the robot has to change when interacting with either single customers or groups of customers. Giuliani et al. (2013) compared how user groups perceive the robot when it shows only task-based actions or when it also uses social actions. Both user studies used the same instructions for the study participants, they were simply asked to walk up to the robot and to order a drink. An experimenter was visible at all times during both JAMES studies.

#### 2.1.2. JAST, stationary robot with assembly task

The goal of the JAST project was to develop jointly-acting autonomous systems that communicate and work intelligently on mutual tasks in dynamic unstructured environments. The task of the JAST robot was to assemble target objects from a wooden toy construction set together with a human partner. Figure 2B shows the robot. It is the same robot system that was used in the JAMES project. The robot had a table in front of it on which the different assembly parts were laid out. It was able to recognize the objects and to hand them over to the human.

The videos we are using in this work are from the user study reported by Giuliani et al. (2010). The task of the study participants and the robot was to jointly construct two target objects. In the experiment, the authors compared two different strategies for generating referring expressions to objects, a traditional strategy that always generated the same expression for the objects and an adaptive strategy that made use of the situated context knowledge of the robot. The participants worked together with the robot in one-on-one interactions. During the study, participants were not able to see the experimenter, who was sitting behind a poster wall.

#### 2.1.3. IURO, mobile robot asking for directions

The goal of the IURO project was to develop a robot that navigates and interacts in densely populated, unknown human-centred environments and retrieves information from human partners in order to navigate to a given goal. The IURO robot was developed to autonomously navigate in an unstructured public-space environment and proactively approach pedestrians to retrieve directions. Figure 2C shows the robot interacting with a pedestrian in the city center of Munich, Germany. The IURO robot was designed with anthropomorphic, but not entirely humanoid appearance. A humanoid head is combined with a functionally designed body. The head is able to produce lip-synchronized speech and express basic facial expressions (Ekman, 1992). Additionally, the robot has two arms, but no hands (to avoid wrong expectations, since the robot is not able to grasp objects). A pointing device for indicating directions is mounted above the robot head.

The videos we used for annotating social signals in error situations were taken from the field trial of the IURO robot in which the final set-up of the robot was validated. To ensure the final robot version running at the best possible set-up, the robot platform was subject to manifold evaluation on different interaction aspects at different points in the project. For a detailed overview on the evaluation set-ups, timeline, and results which led to the final robot prototype, refer to Weiss et al. (2015). The IURO robot interacted with single users and groups of users. During the interactions, the experimenters were mostly, but not always, visible to the participants.

#### 2.1.4. RPBD, mobile robot with kinesthetic teaching

The videos of the last user study that we are evaluating for error situations in this work, were taken from a master's thesis. The goal of this master's thesis was to determine user acceptance factors of robots with different appearances. Specifically, the thesis researches how kinesthetic teaching with an anthropomorphic robot in an industrial context is perceived by users with different backgrounds (programmers vs. naïve users).

The videos we are using in this work are from the user study reported by Stadler et al. (2014). The authors implemented a kinesthetic teaching approach on the humanoid robot platform NAO. The robot was able to record and replay a behavior—a pick-and-place task—taught by the participants. During the experiment, human and robot had direct contact via kinesthetic teaching. Furthermore, the robot was able to recognize and produce speech, and had visual object recognition based on landmark and color detection. Figure 2D shows an experiment participant in interaction with the robot. All study participants interacted alone with the robot, but an experimenter was visible to them at all times.

### 2.2. Video corpus

Our video corpus consists of 201 videos, from which 129 are from the two JAMES user studies, 34 are from the JAST user study, 27 are from the IURO user study, and 11 are from the RPBD user study. We chose only those videos from all user studies that show at least one error situation in which the robot either violated a social norm or had a technical failure. Overall, the videos show 272 individual interactions between a single user or a group of users. The difference between numbers of videos and numbers of interactions is because the videos of the JAST and IURO studies show more than one interaction. The interactions between the study participants and the robots are on average 108.467 s long (standard deviation 47.927 s). During the interactions, 578 error situations occurred in total.

### 2.3. Participants

The videos feature 137 unique study participants, who interacted individually or in groups with the robots. Ninety-four participants were male, 43 were female. Although all experiments took place either in Germany or in Austria, the robot spoke in German with 86 participants, and English with the other 51 participants.

### 2.4. Annotation

**Figure 3:**
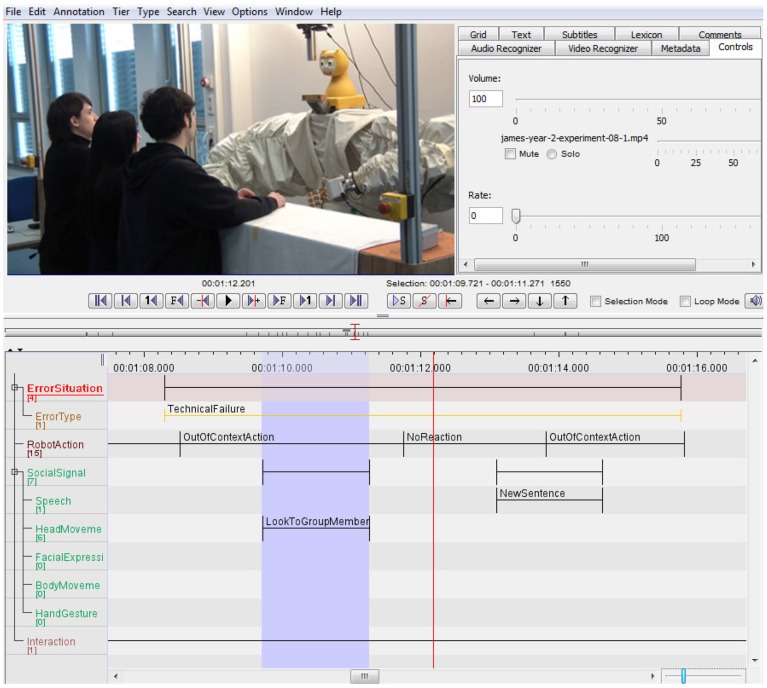
**Screenshot of an error situation in the ELAN annotation tool**.

For data analysis, we annotated our video corpus using the video coding tool ELAN (Wittenburg et al., 2006). Figure 3 shows an example of an ELAN annotation of a video of one of the JAMES user studies using our annotation format.

For annotating the videos, we followed a two-step process. In the first step, we annotated all passages in the videos in which an error in the interaction occurred. For example, when the robot did not understand what a participant had said. We labeled these instances as *error situation*. Since not every error situation is easy to recognize, we coded the error situations in each video file by two independent raters. Afterwards, we calculated the percentage of overlap for the annotated error situations between the two raters. For videos which had less than 75% coding agreement, the two coders looked at the data material again, discussed the differences and reached a consensus on the error situations.

There were two main reasons why coding differed between the two raters. On one hand, one coder annotated the data from a more technical perspective, while the other coder considered the material from a more social viewpoint. For example, if the robot says that it did not understand the study participant it could either mean that the speech recognition module failed (technical perspective) or it could be considered as socially appropriate (social perspective: people sometimes inquire when they do not understand an utterance). From a technical perspective, the utterance would likely be coded as an error situation, while from a social perspective it might be considered as socially acceptable and not an error situation. On the other hand, for correctly identified error situations, the coders did not always agree on when exactly the error situation begins or ends. For example, in case of the bartender robot, one coder started annotating the situation as soon as the robot hand moved toward the bottles, whereas the other coder only started after it was clear that the robot would actually grasp the wrong bottle. At the end, the annotators agreed that all codings should be done from the viewpoint of the study participant, which means that in the example the error situation would start from where the participant can see that the robot will grasp the wrong bottle.

In the second coding step, we annotated the actions the robot performed during the error situations, together with the social signals the study participants exhibited at the same time. For annotation of the social signals we used the following five classes, that we chose in order to be able also to annotate social signals that occur in parallel:

- *Speech*: verbal utterances by the participants, including *task-related sentences* for the task given in the user study, *questions* that the participants ask to the robot, a group member or the experimenter, and *statements* the participants make.
- *Head movements*: instances where the participant *looks* to the robot, a group member, or the experimenter. Head movements can also be *nodding*, *shaking*, and *tilting* the head.
- *Hand gestures*: movements that participants make with their hands, including *pointing* gestures, *emblems*, instances where the participants *manipulate an object*, or when they *touch* themselves on the body or in the face.
- *Facial expressions*: expressions as for example *smiling* that can be observed on the participants' faces. These also include signals like *rising the eyebrows* or making a *grimace*.
- *Body movements*: all movements that the participants make with their whole body, including *leaning* toward or away from the robot, *moving* toward or away from the robot, and *changes in body posture*.

We annotated the social signals that occur during the error situations in our videos according to these five classes. In the next section, we present the results of these annotations.

## 3. Results

Table 1 shows an overview of all annotated verbal and non-verbal social signals that occurred during the error situations in our study videos. In the category **head movements** we found that participants often look back and forth between robot and experimenter or a group member if present. Depending on the study task, they also look back and forth between the robot and objects in front of them. The participants also sometimes nod, shake, or tilt their head. We annotated 947 items in the **speech** category. We subdivided the speech utterances into task-related sentences, sentences that the study participants said to the robot to move the given task forward, statements that participants made to comment on the situation to either the robot or another human, questions that participants asked to the robot or a human, audible laughter, and other utterances, for example attempts to speak or hesitation sounds. One participant whistled at the robot to get its attention. In the category **facial expressions**, we found that participants often smiled in error situations. Sometimes the participants grimaced, for example when they showed a concerned look or pouted. Quite often, the participants raised their eyebrows. When interacting with the robot, we found that participants mostly stand still and do not show much **body movement**. For the majority of body movements, participants leaned toward or away from the robot, less often they completely stepped away from the robot or changed their posture. In comparison to other social signals, we found only a few **hand gestures**. Participants often touched themselves in the face or put their hands on the hips. If they had an object in reach, they manipulated that object. Pointing gestures and iconic gestures were quite rare, we annotated only 1 thumbs up gesture and 9 persons, who waved at the robot. Other hand gestures include for example drumming with the fingers on a surface, raising one or both hands, and making a fist with the hand.

**Table 1:** **Counts for all annotated social signals in the categories head movements, speech, facial expressions, body movements, and hand gestures**.

**Head movements**	**1279**	**Speech**	**947**	**Facial expressions**	**484**
Look at robot	434	Task-related sentence	487	Smile	314
Look at experimenter	230	Statement	170	Grimace	124
Look into a direction	230	Question	111	Raise eyebrows	46
Look at group member	151	Laugh	98		
Tilt head	83	Correction	20		
Look to object	72	Other	61		
Nod	40				
Shake head	39				
**Body movements**	**272**	**Hand gestures**	**248**		
Lean	191	Touch own body	45		
Move	33	Manipulate object	61		
Other	48	Pointing	21		
		Emblem	10		
		Other	111		

Next, we performed a statistical analysis to compare the differences in shown social signals for three dependent variables: *social norm violation* vs. *technical failure*; *experimenter visible* vs. *experimenter not visible*; and *group interaction* vs. *single user interaction*. For that, we first performed an analysis of our data and found that all variables are not normally distributed. Therefore, we chose to compare the data populations with a Wilcoxon–Mann–Whitney test. Furthermore, we extracted the data for each error situation individually from the annotations in order to be able to group them by the dependent variables. The error situations had an average duration of 18.314 s (standard deviation 20.861 s). These numbers indicate that many of the error situations are either quite short or last very long. From the 578 annotated error situations, 427 are social norm violations and 151 are technical failures, in 420 error situations the experimenter was visible and in 158 situations the experimenter was not visible, and we annotated 257 group interactions and 321 single user interactions.

Table 2 shows the result of the Wilcoxon–Mann–Whitney test for the dependent variable *social norm violation* vs. *technical failure*. We only show the social signals for which we found statistically significant results. The results show that study participants more often smile and laugh audibly during technical failures than during social norm violations. The participants more often look back and forth between the robot head and objects in front of them during social norm violations. In contrast to that, they look more often to the experimenter during technical failures. The other statistically significant differences we found fall into the range of verbal social signals. During social norm violations, the participants in general speak more, they say task-related sentences to the robot and also repeat these sentences more often than during technical failures. However, during technical failures, the participants comment the situation more often and make statements to group members.

**Table 2:** **Social signals shown during social norm violations and technical failures**.

**Social signal**	**Social norm violation**	**Technical failure**	**Wilcoxon–Mann–Whitney**	
	**Mean (std)**	**Mean (std)**	***p*-value**	**W**
Laughter	0.10 (0.42)	**0.33 (1.02)**	<0.0001	28346.0
Smile	0.46 (0.97)	**0.69 (1.03)**	0.0004	26997.5
Look to robot head	**0.53 (1.39)**	0.35 (1.64)	0.0009	36378.5
Look to object	**0.17 (0.73)**	0.03 (0.18)	0.0308	33934.0
Look to experimenter	0.27 (0.71)	**0.54 (1.21)**	0.0124	29161.5
Say task-related sent.	**0.37 (0.70)**	0.24 (1.00)	0.0012	36552.5
Repeat task-related sent.	**0.36 (1.12)**	0.26 (0.98)	0.0026	35808.5
Make statement to group	0.04 (0.27)	**0.11 (0.41)**	0.0079	30524.5

We annotated 427 social norm violations and 151 technical failures. We present for each social signal the mean number of occurrences per interaction and the standard deviation in parenthesis. Higher results are marked in bold.

Table 3 shows the result of the Wilcoxon–Mann–Whitney test for the dependent variable *experimenter visible* vs. *experimenter not visible*. We only present the social signals for which we found statistically significant results. The results show that the study participants display much more non-verbal social signals when the experimenter is visible, for example tilting the head, making hesitation sounds, smiling, laughing audibly, nodding, and leaning back. Overall, the participants also talk more when the experimenter is visible, they say more task-related sentences to the robot, make more statements, and ask more questions. In contrast to that, the participants more often look back and forth between robot hand, robot head, and objects in front of them when the experimenter is not visible.

**Table 3:** **Social signals shown when the experimenter is visible during an interaction or not**.

**Social signal**	**Exp. visible**	**Exp. not visible**	**Wilcoxon–Mann–Whitney**	
	**Mean (*SD*)**	**Mean (*SD*)**	***p*-Value**	***W***
Tilt head	**0.16 (0.52)**	0.05 (0.29)	0.0048	30636.0
Make sound	0.08 (0.50)	**0.09 (0.33)**	0.0468	34547.5
Smile	**0.56 (1.00)**	0.41 (0.95)	0.0342	30063.5
Laughter	**0.22 (0.74)**	0.01 (0.08)	<0.0001	28720.0
Raise eyebrows	0.06 (0.29)	**0.14 (0.43)**	0.0038	35322.0
Nod	**0.08 (0.32)**	0.02 (0.14)	0.0345	31748.5
Lean back	**0.07 (0.28)**	0.01 (0.08)	0.0038	31255.5
Attempt to take object	0.00 (0.00)	**0.07 (0.80)**	0.0212	33600.0
Look to experimenter	**0.43 (1.00)**	0.08 (0.32)	<0.0001	27085.5
Look to group	**0.35 (0.91)**	0.00 (0.00)	<0.0001	26860.0
Look to object	0.02 (0.15)	**0.44 (1.15)**	<0.0001	39812.5
Look to robot hand	0.14 (0.88)	**0.63 (1.76)**	<0.0001	38557.5
Look to robot head	0.19 (1.00)	**1.27 (2.07)**	<0.0001	46870.5
Say task-related sent.	0.32 (0.85)	**0.39 (0.63)**	0.0159	36427.5
Make statement to robot	**0.09 (0.51)**	0.01 (0.11)	0.0397	31933.0
Make statement to group	**0.09 (0.36)**	0.00 (0.00)	0.0011	31047.0
Make statement	**0.13 (0.47)**	0.03 (0.16)	0.0044	30840.0
Question to experimenter	**0.11 (0.43)**	0.01 (0.11)	0.0016	30748.0
Question to group	**0.04 (0.26)**	0.00 (0.00)	0.0255	32153.0
Question to robot	**0.05 (0.36)**	0.00 (0.00)	0.0255	32153.0

The experimenter was visible in 420 and not visible in 158 situations. We present for each social signal the mean number of occurrences per interaction and the standard deviation in parenthesis. Higher results are marked in bold.

Finally, Table 4 shows the result of the Wilcoxon–Mann–Whitney test for the dependent variable *group interaction* vs. *single user interaction*. Similar to the variable experimenter visible/not visible, we found that the participants show much more non-verbal signals, when interacting in groups with the robot. They laugh audibly, smile, and tilt their heads more often, when in an error situation. The participants look more often to the experimenter or a group member, when in a group interaction, but they look more often back and forth between the robot and an object, when they interact alone with the robot. We also found that the participants say more task-related sentences and make more statements commenting the situation when they are in a group. However, the participants ask more questions to the experimenter when they are in a single interaction with the robot.

**Table 4:** **Social signals shown during single user and group interactions**.

**Social signal**	**Group**	**Single user**	**Wilcoxon–Mann–Whitney**	
	**Mean (*SD*)**	**Mean (*SD*)**	***p*-Values**	***W***
Tilt head	**0.20 (0.57)**	0.07 (0.37)	0.0002	45010.5
Smile	**0.71 (1.14)**	0.37 (0.82)	<0.0001	48209.0
Laughter	**0.30 (0.86)**	0.05 (0.35)	<0.0001	47453.5
Change posture	0.02 (0.15)	**0.08 (0.36)**	0.0337	39755.0
Look to experimenter	**0.44 (1.03)**	0.26 (0.73)	0.0030	45377.0
Look to group	**0.57 (1.11)**	0.00 (0.00)	<0.0001	54088.5
Look to object	0.03 (0.20)	**0.21 (0.83)**	0.0002	37957.5
Look to robot head	0.18 (1.19)	**0.73 (1.60)**	<0.0001	31272.5
Say task-related sent.	**0.46 (1.03)**	0.24 (0.51)	0.0075	45263.5
Rephrase task-related sent.	**0.22 (0.94)**	0.04 (0.28)	0.0006	43924.5
Make statement to group	**0.14 (0.45)**	0.00 (0.00)	<0.0001	45582.0
Make statement to robot	**0.14 (0.64)**	0.01 (0.11)	0.0002	43800.0
Make statement	0.06 (0.38)	**0.13 (0.44)**	0.0068	38765.5
Attempt to speak	**0.05 (0.23)**	0.01 (0.11)	0.0226	42502.0
Question to experimenter	0.05 (0.25)	**0.12 (0.44)**	0.0456	39535.5
Question to group	**0.06 (0.32)**	0.00 (0.00)	<0.0001	43335.0

We annotated 257 group interactions and 321 single user interactions. We present for each social signal the mean number of occurrences per interaction and the standard deviation in parenthesis. Higher results are marked in bold.

After the presentation of the results, we now discuss their meaning and the implications for HRI in the following section.

## 4. Discussion

While annotating, we found that the difference in change of behavior during error situations and non-error situations is sometimes visible even in single user study instances. For example, during one of the studies of the JAMES project (Giuliani et al., 2013), one of the study participants had already ordered a drink and watched a group member ordering his drink. The bartender robot did not understand the other group member and repeatedly kept asking for the order. Because of this, the participant repeatedly had to smile and even sometimes laughed audibly. He furthermore kept looking back and forth between the other group member and the robot. This behavior changed completely as soon as the experimenter resolved the situation by declaring that there was an error with the system. Following this statement, the experiment participant did not smile any more and kept looking to the experimenter, although the robot kept asking for the order. This instance clearly shows how the behavior of the participant changed in seconds when coming from a social norm violation to a technical failure.

The counts of social signals in our annotations, that we show in Table 1, reveal three interesting results: firstly, there were many examples for error situations in which participants kept looking back and forth between robot and a group member, or robot and experimenter, or robot and objects in front of them. This is an indicator that the experiment participants are quite literally “looking” for a solution to resolve the error situation. The recognition and analysis of head movements are typically not part of the input modalities of human–robot interaction systems. Our results suggest that developers of input modalities for HRI systems should also look into expanding into this direction. Secondly, we found that participants do not use many hand gestures during error situations. Furthermore, the majority of hand gestures do not fall into the categories that typically are studied in gesture communication. We found only a few pointing gestures and emblems, which questions the importance of these gesture categories for human–robot interaction. Also, we argue that the hand gestures that fall into the categories *touch own body*, *manipulate object*, and *other*, are not used by the participants to communicate their intentions. Thirdly, the participants often smiled during error situations, more often when they experienced technical failures and less often during a social norm violation. Work by Hoque et al. (2012) shows that humans smile in frustrating situations. They recorded the faces of participants who filled out a web form that was designed to elicit frustration. 90% of the participants smiled in these frustrating situations. We have no subjective data that could tell us whether the participants experienced frustration during the error situations with our robots. However, our video analysis indicates that they were frustrated, even more in the case of technical failures than when experiencing a social norm violation.

We often observed in the experiment videos that the participants kept standing still without moving at the beginning of an error situation. In psychology literature, this is referred to as “freezing.” It is known that humans stop moving in certain situations. For example, Witchel et al. (2014) showed that the absence of non-instrumental movements can be a sign for the engagement of humans with media. It is also known that humans, as well as animals, freeze as a response to fear or stress (Hagenaars et al., 2014). We argue that the participants in our videos shortly freeze as response to the stress induced by the error situation and the presence of the experimenter. In future work, we will analyse how often, how long, and in which situations the participants kept standing still in our studies.

Our statistical evaluation of the error situations ordered by situation type in Table 2 has to be interpreted with the tasks of the annotated user studies in mind. Of course, the users say more task-related sentences during social norm violations, because they want to solve the given task during the study. For example, many of the task-related sentences are said when the robot does not understand the participant so that the participant has to repeat the sentence. This indicates that speech is the most influential channel to resolve an error situation. However, it is interesting to see that the participants significantly talk less during technical failures.

We believe that the study participants are able to recognize if an error situation is purely technical and, therefore, stop saying task-related sentences to the robot. As we mentioned above, our results also show that the participants smile more often during technical failures, which may be elicited by the frustration they experience (Hoque et al., 2012). The participants also look more often to the experimenter. This suggests that they are looking for guidance from an authority figure during a situation they did not experience before (Smith et al., 2014).

Finally, the results of the statistical analysis in Tables 3, 4 in our opinion contain the most interesting result of our analysis. The participants show far more non-verbal signals, when they are in a group and/or can see the experimenter. This suggests that participants do not see the robot as an interaction partner that can interpret the same signals as a human. This is also supported by the fact that the participants make more statements about the error situation when they interact in a group with the robot or when the experimenter is visible. Of course, we also have to mention that some of the results for the conditions *experimenter visible* vs. *experimenter not visible* and *group interaction* vs. *single user interaction* are not surprising. For example, the participants look less often to the experimenter when he/she is not visible, although they still attempt to look at him/her. This serves as a good test for the validity of our annotations.

## 5. Conclusion

Our video analysis of social signals in error situations during human–robot interaction experiments shows three main results. (1) The participants use head movements as a social signal to indicate when an error situation occurs. The participants do not use many hand gestures during these situations. Furthermore, the participants often smile during error situations, which could be an indication for experienced frustration. (2) The participants try to resolve social norm violations through speech. They can recognize technical failures of the robot, but they look for guidance by the experimenter in these situations. (3) The participants see the robot as an interaction partner that cannot interpret non-verbal social signals, such as smiling, laughing, nodding, shaking and tilting the head.

These findings have implications for the design and evaluation of HRI systems. *HRI system builders* should consider implementing modules for the automatic detection and interpretation of head movements, especially as an indicator for user engagement or confusion. This modality is not often used as an input channel for robots, but would be fairly easy to implement with modern sensors and image processing technology. It is known that humans use body posture to communicate their intentions (Bull, 1987; Clark, 2003). There is, however, not much work on the interpretation of head movements in particular. The importance of head movements is also supported by research from the cognitive sciences and neuropsychology that shows that head movements play a vital role in recognizing faces (O'Toole et al., 2002), especially for patients with congenital prosopagnosia, a condition that makes it difficult for an individual to recognize someone from his or her face (Longmore and Tree, 2013).

*Evaluators of HRI systems* should not discard the data of study trials in which errors occurred, because this data can contain valuable information, as our results show. Our analysis design also shows that the analysis of data from different HRI studies is possible and produces valuable results, when the study data can be coded in abstract categories. Furthermore, when designing the evaluation, one needs to thoroughly consider whether the experimenter or other humans should be present during the study or not, especially when measuring the social signals of study participants toward the robot. Our data clearly shows that the presence of other humans during an HRI study influences the social signals that the participants show. This is also supported by research in psychology, which has shown that study participants change their behavior when they are aware of being recorded (Laurier and Philo, 2006).

In future work, we plan to analyse parts of our video corpus in more detail. Specifically, we will execute a linguistic analysis of the task-related sentences, statements, and questions that the study participants said during the experiment. Furthermore, we will analyse the temporal connection between robot actions and the onset of the reactions of the participants during the error situations. As mentioned in the discussion, we will measure, how often and how long the participants freeze when they experience error situations. Finally, we plan to implement an automatic head movement analysis that interprets the head movements of humans, which is based on the findings of our analysis.

### Conflict of interest statement

The authors declare that the research was conducted in the absence of any commercial or financial relationships that could be construed as a potential conflict of interest.

## Footnotes

^1^A good depiction of human error types can be found at http://www.skybrary.aero/index.php/Human_Error_Types.

^2^http://www.james-project.eu.

^3^http://www6.in.tum.de/Main/ResearchJast.

^4^http://www.iuro-project.eu/.

^5^To be clear: we did not carry out the user studies specifically for this paper. We are revisiting the results from prior studies and analyse them from a different viewpoint in this work.
